# Meeting patients’ health information needs in breast cancer center hospitals – a multilevel analysis

**DOI:** 10.1186/s12913-014-0601-6

**Published:** 2014-11-25

**Authors:** Christoph Kowalski, Shoou-Yih D Lee, Lena Ansmann, Simone Wesselmann, Holger Pfaff

**Affiliations:** Institute for Medical Sociology, Health Services Research and Rehabilitation Science, Faculty of Human Science and Faculty of Medicine, University of Cologne, Eupener Strasse 129, Cologne, 50933 Germany; German Cancer Society, Kuno-Fischer-Straße 8, Berlin, 14057 Germany; Department of Health Management and Policy, The University of Michigan School of Public Health, 1420 Washington Heights, Ann Arbor, 48109-2029 MI USA

**Keywords:** Information needs, Breast cancer, Multilevel modelling, Hospital characteristics

## Abstract

**Background:**

Breast cancer patients are confronted with a serious diagnosis that requires them to make important decisions throughout the journey of the disease. For these decisions to be made it is critical that the patients be well informed. Previous studies have been consistent in their findings that breast cancer patients have a high need for information on a wide range of topics. This paper investigates (1) how many patients feel they have unmet information needs after initial surgery, (2) whether the proportion of patients with unmet information needs varies between hospitals where they were treated and (3) whether differences between the hospitals account for some of these variation.

**Methods:**

Data from 5,024 newly-diagnosed breast cancer patients treated in 111 breast center hospitals in Germany were analyzed and combined with data on hospital characteristics. Multilevel linear regression models were calculated taking into account hospital characteristics and adjusting for patient case mix.

**Results:**

Younger patients, those receiving mastectomy, having statutory health insurance, not living with a partner and having a foreign native language report higher unmet information needs. The data demonstrate small between-hospital variation in unmet information needs. In hospitals that provide patient-specific information material and that offer health fairs as well as those that are non-teaching or have lower patient-volume, patients are less likely to report unmet information needs.

**Conclusion:**

We found differences in proportions of patients with unmet information needs between hospitals and that hospitals’ structure and process-related attributes of the hospitals were associated with these differences to some extent. Hospitals may contribute to reducing the patients’ information needs by means that are not necessarily resource-intensive.

## Background

Breast cancer patients are confronted with a serious diagnosis that requires them to make important decisions. These decisions regard treatment and many aspects of everyday life and require patients to be informed of the advantages and disadvantages of different options. According to the Institute of Medicine [1], “many patients have expressed frustration with their inability to participate in decision making, to obtain information they need, to be heard, and to participate in systems of care that are responsive to their needs” (p. 48f). Previous studies have been consistent in their findings that (breast) cancer patients have a high need for information, especially concerning the severity of their condition and their treatment options [2]. Halkett et al. found that breast cancer patients have a consistently high need for information, which does not significantly decrease over the course of treatment [3]. Mistry et al. came to the same conclusion in a heterogeneous sample of cancer patients [4].

Correlates of information needs have been well described in the literature. Matsuyama et al. found a negative relationship between education level and the information needs of cancer patients [5]. In a study by Beckjord et al. with a heterogeneous sample of cancer patients, cancer survivors who were younger, had comorbid health conditions and had worse physical or mental health had more information needs [6]. Neumann et al. were able to identify five subgroups of patients with different information needs [7]. According to their classification, nearly one-third of their sample fell into the subgroup with no information needs, nearly 40% fell into one of the two subgroups with high psychosocial information needs, and approximately one in six fell into each of the remaining two subgroups, one that only had medical information needs and one with both psychosocial and medical information needs. Thus, one persisting problem is the relatively high proportion of breast cancer patients reporting unmet information needs or dissatisfaction with how their information needs are addressed by their health-care providers [8,9]. The task of providing the right information to each single patient in a way he or she understands clearly is a challenge for each health-care professional and for the treating hospitals.

One of the most important sources of information for breast cancer patients is the hospital in which he or she is diagnosed and/or treated. Breast care centres in Germany, both certified according to the criteria of the federal state of North Rhine Westphalia [10] and those of the German Cancer Society/German Society of Senology [11], are demanded to undertake huge efforts to provide patients with information, be it paper-based or provided verbally by the hospital staff. Little research, however, has been done to investigate differences of information provision between health-care providers. Hence, research in this field using multilevel approaches has been demanded ([12] p.58). Some studies investigated whether provider characteristics were associated with better informed patients e.g. [13,14], but insight into what promotes or hinders information provision on the hospital level is still sparse. Given the substantial evidence that patient involvement in decision making results in improved outcomes, a hospital-level analysis of what contributes to reducing the proportion of patients with unmet information needs is warranted [15-17].

In this study we investigate which hospital characteristics are associated with patients’ unmet information needs. We extend the “conventional set” of structural characteristics employed in most studies, i.e. teaching status, ownership status, and size [18], to include three process approaches that reflect hospitals’ efforts to inform and educate patients: providing patient-specific information material, providing access to self-help groups, and organization of health fairs for patients and families [19-21]. Providing tailored information has been described in the literature as a useful strategy of patient education [14] and the IOM has long recommended tailoring of health information to patients’ needs [22]. Providing access to self-help groups is the second approach investigated. Research evidence on the benefit of self-help group interventions is inconclusive, but a number of studies suggest that self-help groups play an important role in establishing patient-centered care [23] and are associated with improved patient outcomes [24]. The third approach investigated is the organization of information fairs for patients and their family. Information fairs contribute to increasing the accessibility of health information, empowering patients to take charge of their own care, and involving families in decision-making and accommodating their needs as caregivers.

We also consider the “conventional set” of hospital characteristics that reflect the hospital structure– i.e., hospital ownership, patient volume, and teaching status. Hospital ownership may be critical because it determines the allocation of financial and nonfinancial resources in hospitals and thus their ability to meet patients’ information needs [25,26]. Patient volume may influence (unmet) information needs through its impact on clinical workload, coordination, and clinicians’ practice experience [27,28]. Teaching hospitals have better access to the latest medical knowledge and the most advanced medical technologies, which may enhance their ability to meet patients’ information needs than non-teaching hospitals [29,30]. On the other hand, teaching hospitals have multiple missions and a more complex organizational structure that may increase the difficulty of coordinating the efforts of clinicians to meet the specific information needs of patients [31]. Also considered in the analysis were patient attributes (e.g., age, education, cancer stage) that may affect (unmet) information needs.

## Methods

We employed a cross-sectional study design. Data were collected from two sources: one was a postal survey of newly diagnosed breast cancer patients treated in German breast cancer center hospitals and the other a postal survey of hospital key informants in those breast cancer center hospitals. The participating hospitals were accredited by the German Cancer Society/German Society for Senology [11]. Both surveys received ethical approval from the Ethics Committee of the Medical Faculty of the University of Cologne, Germany.

### Patient survey

A patient survey was conducted in 2010 in 160 of the 251 certified breast cancer center hospitals. Hospitals participating in the survey were similar to non-participating hospitals in ownership, teaching status, and patient volume. The overall purpose of the survey was to compare the quality of healthcare provided in participating breast cancer center hospitals as perceived by patients with a special focus on patient-centeredness and information provision. Details on the survey have been reported elsewhere [8]. Patients undergoing treatment for primary breast cancer in one of the breast cancer center hospitals were invited to self-administer a questionnaire at home after being discharged from the hospital. Patients were included if they: (1) had undergone inpatient surgery between March 22nd and November 31st, 2010, for newly-diagnosed breast cancer, (2) had at least one malignancy, and (3) had at least one postoperative histological evaluation. Before being discharged, eligible patients were asked to give written consent to participate in the survey. Of the 9,354 patients who were eligible, 8,226 consented to the survey and made up the sample of potential respondents. The questionnaire was sent out to those patients within one week of receiving written consent. The survey was designed according to Dillman’s Total Design Method, with three contact attempts being made [32]. A total of 7,301 patients responded, with a response rate of 88.8% of the consenting patients. Survey data were supplemented with clinical data provided by the hospitals.

### Hospital key informant survey

A survey of key informants in breast cancer center hospitals that participated in the patient survey was conducted in 2011 to collect information on hospital structures and activities to strengthen patient-centeredness. The Questionnaire for Breast Cancer Centers Key Informants “FRITZ” [33], along with a letter introducing the study, were sent to one contact in a managerial position within a breast cancer center hospital, i.e., the hospital director/manager or his/her appointed deputy. The key informant was asked to fill out the questionnaire or to pass it on to another individual who was qualified to respond to the survey because of his/her familiarity with the subject matter. Details on the procedure and results of the survey were reported elsewhere [34]. Of the 160 breast cancer center hospitals that participated in the patient survey, 111 returned the key informant survey (69.4% response rate). Patient disease characteristics did not vary significantly between hospitals that provided key informant data and those who did not, but hospitals that responded to the key informant survey tended to have better patient survey results [35]. This was especially true for satisfaction with care items, which were not analyzed in the current study. No differences were found between responding and non-responding hospitals in terms of size, ownership, and teaching status.

### Measures

#### Dependent variable

Unmet health information needs: The patient survey included nine questions that asked patients whether, during their hospital stay, they would have liked to receive more information on topics of vital interest to them (Table 1). The questions were designed based on expert consensus and have been adopted in surveys of accredited breast cancer centers in Germany since 2005 and reported to hospitals in benchmark reports ever since [36]. The questions were also used in studies on information needs among cancer patients [7]. For each of the survey questions, the response options were “yes”, “no”, and “don’t know”. We coded “yes” to 1 and “no” to zero. About 8% of respondents answered “don’t know” to one or more of the items and those answers were treated as missing. A factor analysis revealed a single-factor structure that underlay those nine survey items. Thus, we summed the answers as a composite indicator (value = 0 to 9) to represent unmet information need, with a higher value representing greater unmet information needs. In calculating the composite indicator, we adjusted the score to the 0–9 range for respondents with at least five valid answers. The Cronbach’s alpha of the composite indicator was 0.81, indicating high internal consistency.

**Table 1:** **Proportions of patients indicating unmet information needs (hospital minimums and maximums) in breast cancer center hospitals**

**Would you have liked to receive more information on…**	**Proportion** **(hospital min-max)**	**n**	**Don’t know**
… healthy lifestyles (nutrition, alcohol, smoking, etc.)?	26% (4%-50%)	4,637	291
… physical and mental strains in everyday life?	39% (8%-62%)	4,586	319
… self-help groups?	15% (0%-39%)	4,592	313
… books and brochures about your illness?	13% (0%-36%)	4,671	236
… health-promoting measures?	42% (9%-71%)	4,643	252
… help and support at home?	22% (0%-39%)	4,503	381
… psychosocial/psychological support?	19% (3%-50%)	4,557	318
… rehabilitation possibilities?	33% (6%-75%)	4,707	205
… help for daily activities (wigs, household chores)?	19% (0%-39%)	4,507	381
	Mean (SD)	n	range
Unmet information score	2.32 (2.55)	4,809	0-9

#### Independent variables

Hospital structural characteristics: Three structural characteristics of hospitals were examined: ownership status (public; charitable; and for-profit), patient volume (annual number of surgeries on breast cancer patients, grouped into: ≤100; 101 to 200; 201 to 300; 301 to 400; and >400), and teaching status (yes; no).

Hospital process characteristics: Three approaches of hospitals were investigated that reflect the hospital’s effort to inform their patients: provision of patient-specific health information, provision of access to self-help groups, and organization of patient and family health fairs. These approaches were measured, respectively, by the following questions in the hospital key informant survey: “Does the hospital provide health information materials that are tailored to the patient’s disease condition? (yes; no)”, “Does the hospital make sure that patients have access to self-help groups in the hospital? (yes; no)”, and “Does the hospital organize information events for patients and families? (yes, on a regular basis for either patients or family or both; no, not on a regular basis)”.

Patient attributes: Five socio-demographic variables from the patient survey were included in the analysis: age, education attainment (no education certificate achieved; lower secondary school; intermediate secondary school; technical college/university entrance certificate), living with a partner (yes; no), insurance status (solely statutory health insurance (SHI); SHI plus additional voluntary private insurance/private insurance), and native language (German; other). Before the reunification two different education systems existed in Germany. The categorization was done according to years of schooling and excludes tertiary education. The health insurance is a proxy indicator for social status, with higher income groups being more likely to be privately insured or to have SHI with additional voluntary private insurance. In addition to patients’ socio-demographic attributes, we also obtained clinical and treatment information on patients from the participating hospitals. The information included cancer stage using UICC categories [37] (Stages 0 to IV), type of surgery (mastectomy; breast conserving treatment) and cancer site (right; left; both).

### Statistical analysis

We performed descriptive and chi-square analyses to examine the prevalence and variation of the three hospital process characteristics that reflect the hospital’s effort to adequately inform their patients.

Breast cancer patients were nested within hospitals. To account for this hierarchical structure, we employed hierarchical linear modelling (HLM) to test the association between hospital attributes and patient unmet health information needs [38]. Four sequential models were estimated. First, we ran a fully unconditional model (FUM/null model) with neither level 1 (patients) nor level 2 (hospitals) predictors to determine the proportion of variance in unmet information needs that was attributable to differences between hospitals. An intraclass correlation coefficient (ICC) was calculated for the FUM to represent the proportion of variance in the dependent variable that was attributable to between-hospital differences. Second, we added patient-level variables (age, cancer stage, cancer site, type of surgery, school leaving certificate, native language, insurance status, and partnership status) to the model. In the third and fourth steps, we added hospitals’ structural and process-related characteristics to see if the addition of each group of the variables improved the model fit. HLM 7 software was used for inferential analyses and SPSS 21.0 was used for descriptive and bivariate analyses.

No imputation was performed for missing data. Patient observations with missing information on the dependent variable were excluded. Missing data on the independent variables were included in the model as separate categories to avoid case deletion, and omitted in the results table.

## Results

As seen in Table 1, which presents the average percentages (as well as minimums and maximums) of patients reporting unmet information needs, there remained unmet information needs in German breast cancer center hospitals and the degree of patient unmet information needs varied across hospitals as well as information topics. The four areas with highest unmet information needs were in relation to health promotion activities, physical and mental strains in daily life, rehabilitation services, and healthy lifestyles.

Table 2 presents the descriptive results of the independent patient level variables for those 5,024 patients from hospitals that also participated in the key informant survey. Two thirds of patients were between 50 and 69 years old and less than a quarter had a high school diploma qualifying for university or technical college. Close to 20% had some form of private insurance. Less than 4% spoke German as their second language. Roughly three quarters lived with a partner. With regard to disease and treatment characteristics, about one sixth of patients were in an advanced stage of cancer (stage III or IV); approximately 75% received breast-conserving treatment; the very majority of patients had cancer on one side.

**Table 2:** **Patient characteristics (n = 5,024)**

	**Valid percent (n)**
Age	
18-39	4.1 (204)
40-49	17.1 (850)
50-59	27.7 (1,378)
60-69	30.1 (1,500)
≥70	21.0 (1,044)
Missing	(48)
Highest education achieved	
No education certificate achieved	2.2 (107)
Lower secondary school (8 or 9 yrs)	41.4 (2,003)
Intermediate secondary school (10 yrs)	34.0 (1,642)
High school certificate (12 or 13 yrs)	22.4 (1,081)
Missing/other	(191)
Health insurance	
SHI only	81.8 (4,026)
Private/SHI + voluntary additional insurance	18.2 (898)
Missing/other	(100)
Living with a partner	
Yes	73.3 (3,644)
No	26.7 (1,330)
Missing	(50)
Native language	
German	96.2 (4,773)
Other	3.8 (187)
Missing	(64)
Stage	
Stage 0	6.2 (264)
Stage I	45.6 (1,995)
Stage II	33.9 (1,453)
Stage III	10.6 (454)
Stage IV	3.7 (160)
Missing	(738)
Type of surgery	
Mastectomy	25.4 (1,219)
Breast conserving treatment	74.6 (3,573)
Missing	(232)
Cancer site	
Left	50.1 (2,437)
Right	47.2 (2,299)
Both	2.7 (129)
Missing	(158)

Results showed substantial variation among German breast cancer center hospitals in their efforts to adequately inform their patients (Table 3). Of the approaches, organization of patient and family health fairs was prevalent in 90 hospitals (81.1%) in the sample. Forty (36.0%) hospitals provided information material that was tailored to the specific patients. Only three hospitals (2.8%) provided breast cancer patients no access to self-help groups. No significant associations were found between the structure and process attributes except for providing specific information material that happened more often in teaching than in non-teaching hospitals.

**Table 3:** **Prevalence of hospitals’ efforts relating to meeting patient-information needs**

	**Patient-specific information material**			**Access to self-help groups provided**			**Health fairs for patients/families**		
	**Yes**	**No**	**X** ^**2**^	**Yes**	**No**	**X** ^**2**^	**Yes**	**No**	**X** ^**2**^
All hospitals	40 (36%)	71 (64%)		105 (97%)	3 (3%)		90 (81%)	21 (19%)	
Ownership status									
Public	15 (27%)	41 (73%)	4.21	56 (100%)	0 (0%)	4.41	49 (88%)	7 (12%)	4.28
Charitable	13 (45%)	16 (55%)		27 (96%)	1 (4%)		20 (69%)	9 (31%)	
For-profit	12 (46%)	14 (54%)		22 (92%)	2 (8%)		21 (81%)	5 (19%)	
Patient volume									
≤ 100	5 (38%)	8 (62%)	8.39	13 (100%)	0 (0%)	2.54	10 (77%)	3 (23%)	3.46
101 to 200	13 (25%)	38 (75%)		49 (98%)	1 (2%)		41 (80%)	10 (20%)	
201 to 300	12 (39%)	19 (61%)		28 (93%)	2 (7%)		26 (84%)	5 (16%)	
301 to 400	6 (75%)	2 (25%)		8 (100%)	0 (0%)		5 (63%)	3 (38%)	
> 400	3 (50%)	3 (50%)		6 (100%)	0 (0%)		6 100%)	0 (0%)	
Teaching hospital									
Yes	38 (40%)	57 (60%)	4.49*	90 (98%)	2 (2%)	.84	76 (80%)	19 (20%)	.50
No	2 (13%)	14 (88%)		15 (94%)	1 (6%)		14 (88%)	2 (13%)	

N; percentages; note: We used pair-wise deletion in the chi-square analysis. Two hospitals had no information on patient volume and three had no information on access to self-help groups. Pearson’s chi-square and Fisher’s exact test yielded the same results for statistical significance; *< .05.

Results of the hierarchical linear model are presented in Table 4. The ICC of the fully unconditional model is 0.030, suggesting that between-hospital differences accounted for a small amount of variance in patient unmet information needs and that the variation occurred primarily at the patient level. Indeed, most of the patient-level variables examined in the study are statistically significant in explaining patient unmet information needs (model 1). Patients with the following attributes appeared to have greater unmet information needs: undergoing mastectomy, being younger, having statutory insurance coverage (lower income), not living with a partner, and speaking German as a second language. No statistical significance was found for cancer stage, education, and cancer site (not reported in the table for the sake of parsimony).

**Table 4:** **Results of the hierarchical linear regression models on unmet information needs**

	**Model 1**	**Model 2**	**Model 3**
**Intercept**	1.82***	1.53***	1.68***
**Patient characteristics**			
Mastectomy	.23*	.23*	.23**
Statutory health insurance	.18*	.18*	.19*
Not living with a partner	.37***	.36***	.37***
Age groups (ref. 60 to 69)			
≤ 39	.64***	.63***	.62***
40 to 49	.40**	.41**	.40**
50 to 59	.42**	.43***	.43***
≥ 70	-.47***	-.47***	-.47***
Native language other than German	.82***	.82***	.81***
**Hospital characteristics - structure**			
Teaching		.23	.32**
Patient volume (ref. 101 to 200)			
≤ 100		-.09	-.05
201 to 300		.41**	.45**
301 to 400		-.07	.09
≥ 401		.04	.21
Ownership (ref. for-profit)			
Public		-.01	-.11
Charitable		-.11	-.18
**Hospital characteristics – process**			
Patient-specific information material			-.39**
Access to self-help groups			.18
Health fairs for patients/family			-.26*
*Variance components for random effects:*			
Between-hospital variance (τ_00_); SD	.20; .44***	.16; .40***	.14; .37***
Degrees of freedom	110	98	98
Chi-square	262.91	212.21	185.16
ICC (FUM: .030)	.032	.025	.022

Models adjusted for additional patient characteristics (stage, cancer site, education); n = 4,809 patients; N = 111 hospitals; *p < .05; **p < .01; ***p < .001.

Addition of hospital structural and process characteristics (models 2 and 3) improved the model fit and led to a reduction of the ICC (0.025, 0.022, respectively). Based on the change in ICC, those variables contributed to reducing the amount of unexplained variance on the hospital level by over 30%. Two of the three process characteristics – provision of patient-specific health information and organization of patient and family health fairs – had a negative and statistically significant coefficient, suggesting that they contributed to reducing unmet patient information needs. No association was found for providing access to self-help groups.

Among the hospital structural characteristics, teaching status was significantly positively associated with unmet information needs. Higher patient volume appeared to be associated with higher unmet information needs.

## Discussion

Consistent with previous research, substantial proportions of breast cancer patients reported unmet information needs in various areas. In this study we investigated associations of unmet information needs with hospital-level structure and process characteristics, in addition to patient-level attributes.

In our analysis, most of the variation in patient unmet information needs was accounted for by breast cancer patients’ socio-demographic and disease characteristics. These findings are consistent with the literature. Finney Rutten et al. [2], for example, found in their review that age was negatively associated with seeking information among cancer patients. Veloso et al. [39] observed that higher information needs tended to occur in patients with less resources, including social and familial support. A patient characteristic that has not been broadly discussed in the literature and is shown to be strongly associated with unmet information needs in this study is the primary language of the patient. We found breast cancer patients that spoke German as a second language were more likely to have unmet information needs. This finding, though not surprising, points to an increasing challenge of health care providers in developed countries in meeting the needs, including health information needs, of immigrant patient populations [40,41].

The ICC of the hierarchical linear modeling was relatively small, suggesting that between-hospital differences (i.e., hospital-level factors) contributed to explaining the variance in patient unmet information needs only to a little extent. In Germany, breast cancer centers were established with the aim to reach a consistently high level of care quality. This may explain the small ICC in the analysis. In fact, the ICC in our analysis was comparable with those reported in previous research and analyses that show a high ICC in relatively standardized health care settings are rare. Sjetne et al., for example, reported ICCs ranging from 0.002-0.065 for several patient experience indicators [31].

We, however, found that breast cancer patients treated in teaching hospitals were significantly more likely to report unmet information needs. The research literature has reported inconsistent results regarding the performance of teaching hospitals perform in different areas. Several studies found that, in comparison to non-teaching hospitals, teaching/academic hospitals achieved better results in process and, especially, outcome aspects of clinical care quality [42-45]. Landon et al. found teaching status to be associated with better diagnosis and treatment performance, but worse patient counselling [46]. An explanation of these various results is that teaching hospitals have multiple missions and that they have difficulties simultaneously maintaining excellence in clinical care, teaching and research, and successfully meeting patients’ information needs.

We in addition found that two of the approaches that reflect the hospitals’s effort to inform and educate their patients – provision of patient-specific health information material and organization of patient and family health fairs – were associated with smaller unmet information needs. Epstein & Street [12] and the IOM [1] called for research to examine the organizational context of patient-centered care. Mulcare et al. pointed out that few studies had investigated factors explaining patient information needs [47]. Our study fills these gaps to some extent, but must be interpreted with caution: Neither do we know what specifically was done during health-fairs nor do we know what quality the information material was. In addition, we need to rely on information provided by the key informants’ and cannot exclude over-reporting.

Our findings, however, may have relevant practical implications. Considering the large amount of information a patient might receive during her hospital stay, provision of tailored information that is most relevant to the patients and that meets her specific needs is critical. While tailoring the content of health information to patients is important, it may be equally important to consider the appropriate way that information is delivered to patients – in written form, using graphic display or multi-media presentation, or through oral communication [48,49]. It is reassuring to note that hospitals’ investment in health fairs may be worthwhile. Compared to other hospital-level health activities and interventions such as case management, health fairs are relatively “resource-light”. There is so far little evidence in the literature on the effectiveness of health fairs and specific aspects that make health fairs successful. Neither is there a consensus on the utility of different types of information and educational events for patients, friends, and family. The results reported here need further investigation with respect to both tailoring patient information and organizing health fairs.

We failed to find a significant association between provision of access to self-help groups and patient information needs. There are three likely explanations. First, the approach was implemented in the majority of studied breast cancer center hospitals. Thus, the variation may be too small to detect any significant association. Second, access to self-help groups, unlike the other two “direct contact” approaches [50] to meeting patient information needs, may not be as an effective way to facilitate and manage the flow of useful health information to patients. Third, it could be that self-help groups are more effective in providing emotional and tangible support rather than disseminating health information.

Several research limitations should be considered when interpreting the results of this study. The cross-sectional design of the study made causal interpretations difficult. Furthermore, the results were obtained from a sample of breast cancer patients treated in accredited breast cancer center hospitals in Germany. It is unclear whether the results are generalizable to other patient populations and other health care settings in a different country. We were unable to assess systematic differences in patient participation in the survey. Patients with a low health literacy level and less capable of making informed health care decisions may be less likely to respond to the survey and may have greater unmet information needs. To the extent this is true, we may have under-estimated the degrees of unmet information needs. The same problem of underestimation may occur in patients with more severe breast cancer conditions.

## Conclusions

A fundamental aspect of patient-centered care is patient education and meeting the information needs of patients. It is only when patients are informed that they can become an active partner in the process of their care. We found differences in proportions of patients with unmet information needs between hospitals and that hospitals’ structure and process-related attributes of the hospitals were associated with these differences to some extent. The two attributes that are most easily to change are relatively “low-tech”. Thus, a lesson in our study is that hospitals may contribute to reducing the patients’ information needs by means that are not necessarily resource-intensive. The challenge may be in the shifting of philosophy, attitude, and priority on the part of health care providers.
